# Multimodal Peri-articular Injection with Tranexamic Acid can reduce postoperative blood loss versus Intravenous Tranexamic Acid in Total Knee Arthroplasty: A Randomized Controlled Trial

**DOI:** 10.1186/s13018-021-02685-y

**Published:** 2021-09-03

**Authors:** Hui-ming Peng, Wei Wang, Jin Lin, Xi-sheng Weng, Wen-wei Qian, Wen-da Wang

**Affiliations:** Department of Orthopaedic Surgery, Peking Union Medical College Hospital, Chinese Academy of Medical Sciences & Peking Union Medical College, No.1, Shuaifuyuan Wangfujing, Dongcheng District, Beijing, 100730 China

**Keywords:** Peri-articular injection, Total knee arthroplasty, Tranexamic acid

## Abstract

**Background:**

Tranexamic acid (TXA) has shown significant reductions in blood loss and transfusion rates in total knee arthroplasty (TKA). However, the optimal administration route continues to be debated. The aim of this trial was to compare the effectiveness of intravenous (IV) versus peri-articular injection (PAI) application of tranexamic acid in patients undergoing total knee arthroplasty.

**Methods:**

We conducted a randomized controlled, double-blinded study. A total of 93 patients undergoing primary unilateral TKA were randomly distributed between 2 groups: the IV group (47 cases; 1 g TXA IV) and the PAI group (46 cases; 1 g TXA injected peri-articularly). The amount of total and hidden blood loss (HBL), drainage, transfusion rate, hemoglobin and hematocrit drift, and complications were recorded.

**Results:**

Peri-articular injection of TXA reduced total blood loss (*P* < 0.001) and HBL more than IV use of TXA (*P* < 0.001). No patients in either group received a transfusion. No symptomatic deep venous thrombosis or other severe complications occurred.

**Conclusion:**

Peri-articular injection of TXA significantly reduced total blood loss and hidden blood loss to a greater degree than IV injection in total knee arthroplasty without reduction of drainage volume.

**Trial registration:**

Chinese Clinical Trial Registry, ChiCTR-INR-16010270. Date of registration: December 27, 2016.

## Background

Total knee arthroplasty (TKA) can result in an estimated 800 to 1200 ml of blood loss, which has traditionally resulted in a transfusion rate as high as 53% [1–3]. In addition, the requirement of a blood transfusion has been found to be an independent predictor of peri-prosthetic joint infection after TKA [4]. The management of blood loss during a TKA has been extensively studied [3, 5–8], and one of the most promising agents that have emerged is tranexamic acid (TXA).

TXA is a competitive inhibitor of plasminogen activation that interferes with fibrinolysis improving hemostasis and helping prevent additional blood loss after tourniquet deflation during TKA [9]. This pro-thrombotic effect has raised concern about the risk of thromboembolic complications in total joint arthroplasty. This concern has led to the investigation of topical and local administration of TXA during TKA procedures with continued effectiveness in reducing blood loss and transfusion rates [10].

However, the ideal method of topical or local techniques varies widely from the simple soaking of the wound prior to joint closure to intra-articular injections after deep fascial closure. However, the limited exposure time of TXA with local tissues raises concerns about both simple soaking and intra-articular injections, especially in patients undergoing significant soft tissue releases. Peri-articular injections (PAI) of TXA eliminate the concern of limited local effect and have been shown to significantly improve blood loss and transfusion rates when compared to intra-articular injections [11].

Peri-articular injections aimed at multimodal analgesia are widely used in TKA and have proven to be more effective in providing analgesia compared to traditional methods [12, 13]. To our knowledge, TXA has not been evaluated as a component of multimodal peri-articular injections. We hypothesized that adding TXA as a part of a peri-articular injection containing epinephrine would prolong the local effect of TXA directly on the injured tissue. This may result in reductions in blood loss and transfusion rates comparable to or better than intravenous administration of tranexamic acid but without the systemic toxicity of IV TXA.

Given the above, we evaluated the benefits and complications of an analgesic peri-articular injection with the addition of TXA to a standard IV TXA injection.

## Materials and methods

### Study design

This double-blinded randomized control trial was conducted from January 2017 to July 2018, and the study protocol was approved by our institutional review board and is registered in the Chinese Clinical Trial Registry (ChiCTR-INR-16010270). Written informed consent and research authorization for participation in this study were obtained from each patient before surgery.

Patients presenting during the study time frame undergoing elective unilateral, primary TKA for end-stage osteoarthritis or rheumatoid arthritis were screened for inclusion into the study. Patients were then excluded for any of the following reasons: (1) an allergy to TXA; (2) preoperative hepatic or renal dysfunction; (3) serious cardiac or respiratory disease, including coronary artery stent placement; (4) congenital or acquired coagulopathy, as evidenced by an international normalized ratio (INR) of > 1.4 or a partial thromboplastin time (PTT) of > 1.4 times normal; (5) thrombocytopenia, as identified by a preoperative platelet count of < 150,000/mm^3^; (6) a history of a pro-thrombotic condition; (7) pregnancy; (8) breastfeeding; (9) donated preoperative autologous blood; (10) an age of < 18 years or > 80 years; and/or (11) a preoperative hemoglobin level of < 10 g/dl.

**Fig. 1:**
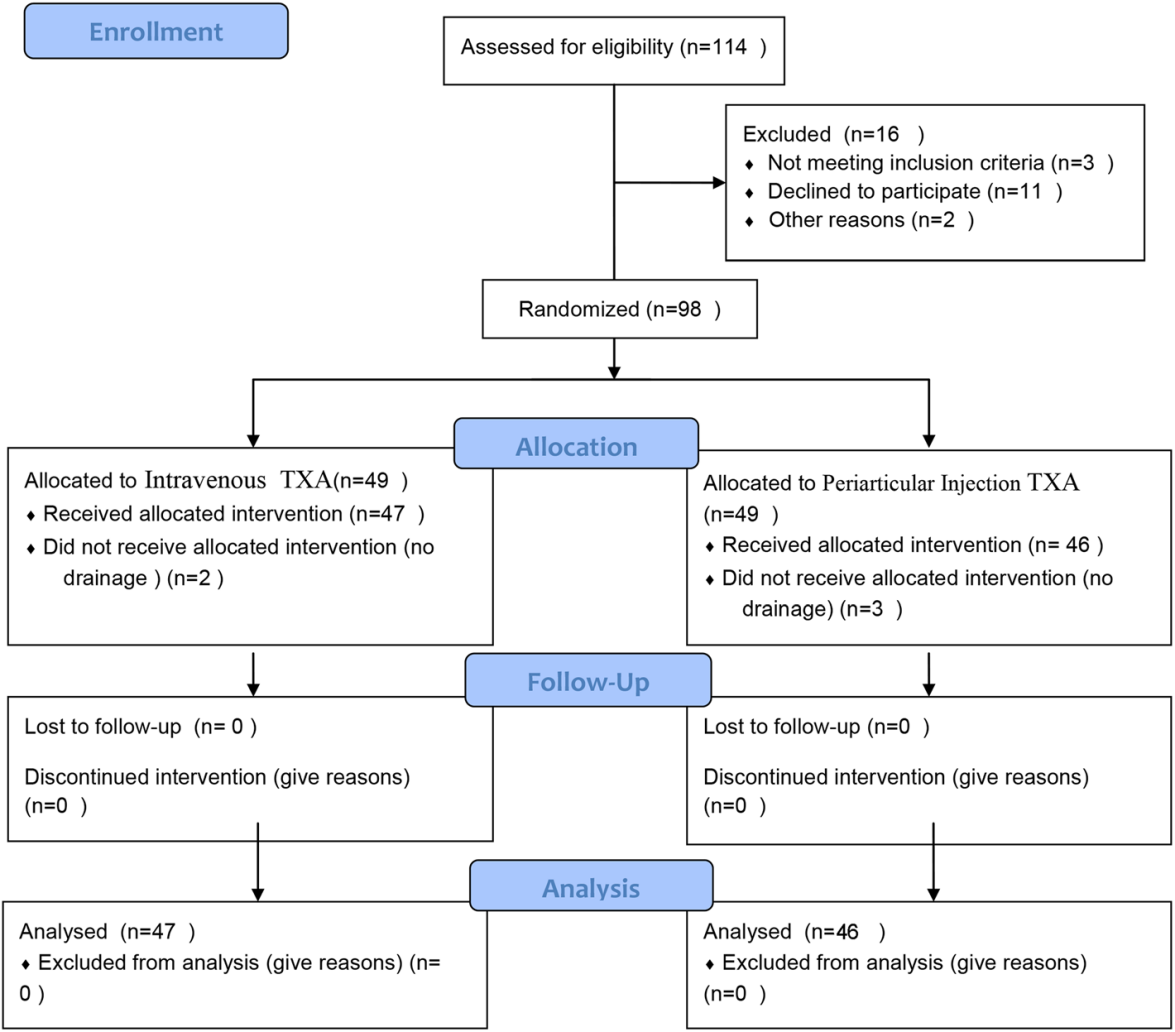
CONSORT 2010 flow diagram

Patients were then randomized to 1 of 2 groups: IV administration of TXA or PAI TXA, in accordance with the random number table. The randomization schedule was generated after the initiation of study enrollment (Fig. 1). The randomization was concealed by sealed, opaque envelopes and was only accessible to the nurse in the operating room who provided the IV and the injection material for each patient. This nurse was not included in data collection or analysis. The surgeons, patients, anesthesiologist, and the data collection team were blinded to randomization.

In the IV group, a 60-ml multimodal cocktail peri-articular injection (MCPI) with ropivacaine, 200 mg/20 ml (AstraZeneca AB, Sweden); morphine, 10 mg/1 ml (domestic company); flurbiprofen axetil injection, 50 mg/5 ml (Beijing Tide Pharmaceutical Co., Ltd., China); adrenaline, 0.25 mg (1:1000); betamethasone, 7 mg/1 ml (Schering-Plow Labo NV, Belgium); and 34 ml of normal saline solution was prepared in three 20-ml syringes. Ten minutes before skin incision, patients received 1000 mg of IV TXA (110 ml total volume) IV administration.

In the PAI group, the 60-ml MCPI was the same as that in the IV TXA group, except that TXA 1000 mg/10 ml (Guangzhou Baiyunshan Pharmaceutical Co., Ltd., China) was added. Before incision, patients received 110 ml of saline IV administration as a placebo.

### Surgical procedures and peri-operative care

All patients underwent TKA under tourniquet control by a single surgeon (Jin Lin, MD). A medial parapatellar approach was utilized, and either a G-II PS (Smith & Nephew, Memphis, USA) or Scorpio (Stryker, Limerick, Ireland) was inserted. No patella was resurfaced during the TKA procedure. The peri-articular injections were performed with the following technique: the first 20 ml of the cocktail was injected into the posterior aspect of the capsule and structures of the knee joint immediately before implanting the prosthesis. After implantation, the remaining 40 ml of the mixture was injected into the extensor mechanism, synovium, anterior capsule, pes anserinus, retinaculum, periosteum, iliotibial band, and collateral ligaments. A drain was placed within the lateral gutter in all patients prior to deep fascial closure.

Postoperatively, a vacuum wound drainage dressing was applied in every patient and a standardized rehabilitation protocol was implemented starting on day 1. In both groups, the drains were removed at 48 h, and the volume of fluid at 24 and 48 h was measured. Chemical and mechanical deep vein thrombosis (DVT) prophylaxis was instituted in all patients and consisted of thromboembolic deterrent stockings along with the daily subcutaneous injection of low molecular weight heparin (0.1 ml/10 kg once a day) until discharge. The criteria for the transfusion of blood products included a hemoglobin level of < 8 g/dl or a hemoglobin level of < 10 g/dl in a patient with symptomatic anemia, or a patient deemed at high risk because of notable underlying cardiac comorbidities. After hospital discharge, patients were seen at 2 weeks, 4 weeks, and 12 weeks postoperatively. They were then seen twice a year or more frequently if complications had occurred.

The primary outcomes measured were the total blood loss (TBL), hidden blood loss (HBL), hemoglobin (HGB) and hematocrit (HCT) drift, and need for a blood transfusion. Hidden blood loss was calculated using the method described by Sehat et al. [14]. HGB and HCT drifts were defined as the difference between preoperative and postoperative minimum values.

Additionally, secondary outcomes included postoperative duplex ultrasounds at post-op days 3 and 14 to detect DVT. D-dimer levels were measured at 24 h postoperatively. Complications were measured up to 3 months postoperatively and included local soft tissue complications, skin necrosis, peroneal nerve palsies, superficial and deep surgical site infections, symptomatic VTEs, cerebrovascular accident, and myocardial infarction.

### Sample size

The study design of this trial was an equivalence test, and the sample size was calculated based on the measured postoperative blood loss. We assumed an alpha error of 0.05 and applied an allocation ratio of 1. A sample size of 37 participants, which allowed for a dropout rate of 10% (4 participants), was calculated to provide 80% power in detecting a difference of 150 ml or reducing postoperative blood loss by 30% in favor of the PAI TXA (from 20 patients of our earlier pilot study); we considered these parameters to be clinically relevant [15].

### Statistical analysis

The Statistical Package for Social Sciences (SPSS Inc, IBM, version 22) was used. For continuous variables, the mean and range were compared between the groups using Student *t* tests. For categorical variables, data was summarized as the frequency and proportion. Proportions were compared between the groups using Fisher’s exact test on univariate analysis.

## Results

A total of 114 unilateral primary total knee arthroplasties were screened for eligibility to be included in the trial. Ninety-eight patients met inclusion criteria and were randomized into the study groups, with each group consisting of 49 patients. Of the 98 patients, five were lost to follow-up, leaving 47 patients in the IV TXA group and 46 patients in the PAI TXA group for statistical analysis. A participant flowchart is provided in Fig. 1.

The mean patient age was 68.2 years (range, 48 to 80 years), and the mean body mass index (BMI) was 27 kg/m^2^ (range, 19 to 38 kg/m^2^). There were no significant differences between the 2 groups for baseline variables including age, sex, medical history, American Society of Anaesthesiologists (ASA) score, BMI, and preoperative laboratory values (*P* > 0.05) (Table 1).

**Table 1:** Demographic data of the patients between two groups

Baseline characteristic	Control (***n*** = 47)	Treated (***n*** = 46)	***P*** value
**Sex (male/female)**	6/41	7/39	0.733
**Age (years)**	68.13 ± 8.12	68.65 ± 9.54	0.776
**Height (cm)**	162.36 ± 6.82	159.87 ± 6.91	0.083
**Weight (kg)**	71.32 ± 9.20	68.65 ± 12.20	0.236
**BMI (kg/m**^**2**^**)**	27.06 ± 3.19	26.81 ± 4.16	0.749
**Diagnosis (OA/RA)**	44/2	44/3	0.651
**Side (left)**	41.30 %	40.43 %	0.931
**ASA score (1/2/3)**	5/21/21	7/27/12	0.171
**Liver function abnormal (%)**	19.57%	14.89%	0.551
**Pre-OP allergy (%)**	15.22 %	10.64%	0.510
**Pre-OP laboratory values**			
**Pre-OP HGB**	132.32 ± 10.25	130.82 ± 10.35	0.486
**Pre-OP HCT (%)**	39.35 ± 3.32	38.77 ± 2.9296	0.375
**Pre-OP D-dimer**	0.70 ± 1.07	1.19 ± 1.70	0.099
**Pre-OP Fig**	2.91 ± 0.55	2.95 ± 0.69	0.788
**Pre-OP WBC**	6.14 ± 1.76	5.64 ± 1.33	0.122
**Pre-OP RBC**	4.31 ± 0.34	4.27 ± 0.33	0.616
**Pre-OP PLT**	235.36 ± 61.04	238.07 ± 73.58	0.847

Notes: *BMI* body mass index, *OA* osteoarthritis, *RA* rheumatoid arthritis*, ASA* American Society of Anaesthesiologists, *HGB* hemoglobin, *HCT* hematocrit, *Fig* fibrinogen, *WBC* white blood cell, *RBC* red blood cell, *PLT* platelet, *OP* operation

### Primary outcome

**Fig. 2:**
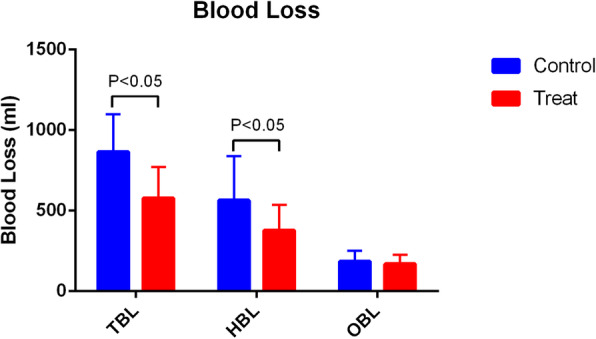
The blood loss of the two groups. The volume was significantly lower for patients in the PAI TXA group compared to the control group (*P* < 0.05)

**Fig. 3:**
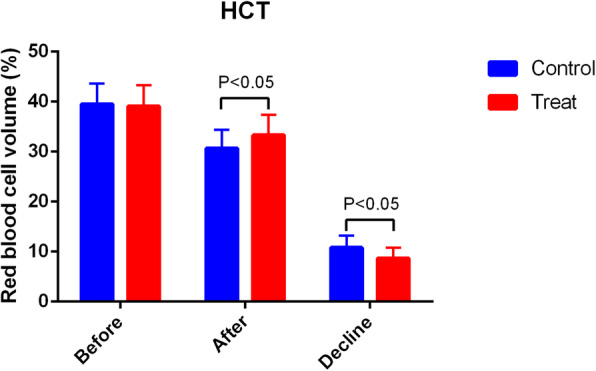
The preoperative and postoperative HCT and HCT drift of the two groups. There was a significant difference (*P* < 0.05)

**Fig. 4:**
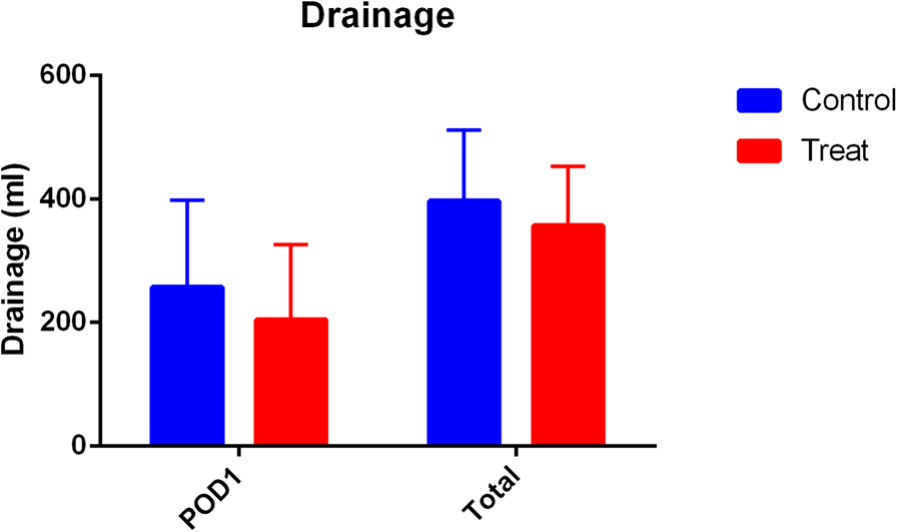
The postoperative drainage volume of the two groups. The volume was lower for patients in the PAI TXA group compared to the control group but there was no significant difference (*P* > 0.05)

**Fig. 5:**
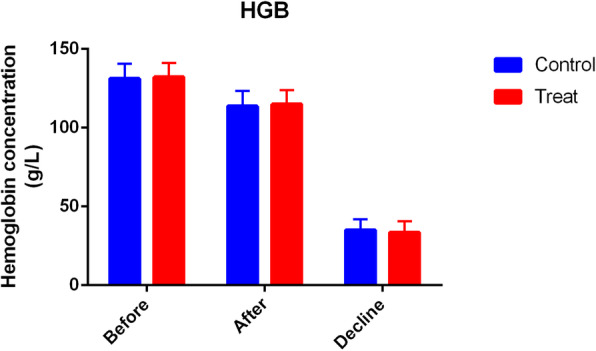
The preoperative and postoperative HGB and HGB drift of the two groups. There was no significant difference (*P* > 0.05)

Patients who received PAI TXA had significantly less TBL and HBL compared with patients who received IV TXA (TBL 641.6 ± 234.0 ml vs 896.0 ± 248.6 ml, *P* = 0.00; HBL 419.8 ± 239.9 ml vs 651.7 ± 243.7 ml, *P* = 0.00) (Fig. 2). The HCT drift on day 3 was significantly less in the PAI TXA group (Fig. 3). The first 24 h drainage post-operation, the total drainage volume, and the HGB drift on day 3 were not significantly different (*P* > 0.05) (Figs. 4 and 5). No patients required a blood transfusion (Table 2).

**Table 2:** Primary outcomes of the patients between two groups

Primary outcomes	Control (***n*** = 47)	Treated (***n*** = 46)	***P*** value
**TBL (ml)**	896.06 ± 248.60	641.64 ± 234.02	0.000
**HBL (ml)**	651.74 ± 243.74	419.85 ± 239.80	0.000
**DBL (ml)**	244.30 ± 60.68	221.79 ± 59.03	0.073
**POD1 drainage (ml)**	255.11 ± 139.82	205.33 ± 117.05	0.066
**Total drainage (ml)**	388.62 ± 121.36	343.59 ± 118.06	0.073
**Tourniquet time (min)**	82.36 ± 4.54	82.93 ± 3.21	0.485
**Anesthesia time (min)**	105.36 ± 4.54	104.30 ± 3.52	0.213
**POD3 HGB**	106.76 ± 13.78	107.41 ± 12.21	0.811
**HGB decline**	26.27 ± 10.17	24.09 ± 9.63	0.290
**POD3 HCT (%)**	30.24 ± 3.65	31.73 ± 3.52	0.048
**HCT decline (%)**	9.73 ± 2.42	7.39 ± 2.80	0.000
**POD1 Fig**	2.98 ± 0.596	3.058 ± 0.698	0.540
**Fig change**	0.0634 ± 0.546	0.111 ± 0.524	0.668
**POD1 RBC**	3.81 ± 0.41	3.84 ± 0.42	0.719
**POD3 RBC**	3.46 ± 0.47	3.51 ± 0.42	0.543
**POD1 PLT**	202.09 ± 53.87	200.98 ± 57.09	0.924
**POD3 PLT**	183.63 ± 50.10	182.74 ± 49.36	0.932
**Transfusion (unit)**	0	0	

Notes: *TBL* total blood loss, *HBL* hidden blood loss, *DBL* dominant blood loss, *HGB* hemoglobin, *HCT* hematocrit, *Fig* fibrinogen, *RBC* red blood cell, *PLT* platelet, *POD* post-operation day

### Secondary outcome

In the IV TXA cohort, 3 (6.3%) of the patients sustained asymptomatic DVT compared with 2 (4.3%) of the patients in the PAI TXA cohort, as confirmed by postoperative ultrasound (*P* = 0.32). The D-dimer values 24 h after operation were not significantly different (*P* > 0.05) (Table 3). None of the patients had clinical evidence of tense hemarthroses, subcutaneous hematomas, peroneal nerve palsies, surgical site infections, skin necrosis, symptomatic VTEs, cerebrovascular accident, myocardial infarction, or deep infection in the short-term (3 months) follow-up.

**Table 3:** Secondary outcomes of the patients between two groups

Secondary outcomes	Control (***n*** = 47)	Treated (***n*** = 46)	***P*** value
**Post-OP DVT**	2/46	3/47	0.632
**Post-OP lower limb ecchymosis**	1/46	7/47	0.00
**POD1 D-dimer**	5.31 ± 3.35	4.41 ± 2.55	0.147
**D-dimer change**	4.61 ± 3.53	3.21 ± 3.04	0.043

Notes: *DVT* deep vein thrombosis, *OP* operation, *POD* post-operation day

## Discussion

Currently, multimodal cocktail peri-articular injections (MCPI) are widely used in peri-operative pain protocols [12, 13]. This study found that the inclusion of TXA into these cocktails was equally efficacious as IV TXA in reducing postoperative blood loss and the need for transfusion. Furthermore, PAI TXA simplified the topical application of TXA and could potentially improve operative times as there is no need for TXA wound soaking. Therefore, with this technique of topical TXA administration, we gain an analgesic effect and reduce postoperative blood loss simultaneously.

The optimal dosage of TXA in peri-articular injections has yet to be determined. Mao et al. found 2000 mg of TXA to be effective in reducing postoperative blood loss during TKA [16]. While Pinsornsak et al. showed a slight increase in the number of blood transfusions when using a 750-mg dose of TXA, which they attributed to a lower preoperative hemoglobin level [17]. In our study, no patients required a blood transfusion while using a dose half of that used by Mao et al. We hypothesize that the use of epinephrine with TXA can provide similar protections while reducing the necessary dose and potentially systemic effects of peri-articular injections.

Good et al. [18] reported that the IV administration of TXA reduced external blood loss but not hidden blood loss after TKA. This is consistent with our results, which showed a significant difference in postoperative hidden blood loss in favor of PAI TXA. IV TXA is a systemic therapy and requires systemic distribution to exert its antibleeding effects, and only a small portion of intravenous TXA solution reaches the target tissue. PAI TXA can act directly on the injured tissue and for a longer duration. As we know that the efficiency of TXA depends on the timing and total dosage in use, in our study, the dosage of TXA is 1000 mg in both groups, but PAI TXA significantly reduced blood loss than IV TXA in TKA, which would be related to the faster action, less waste, and longer action time of PAI TXA. Our results could be clinically significant in preventing blood transfusions, especially in those starting with lower preoperative hemoglobin levels. Unfortunately, due to the small sample size and no transfusions performed, this benefit was not detected in our study.

In this study, there were no symptomatic VTE in either group, and the results of asymptomatic DVT confirmed by ultrasound and D-dimer measurements revealed no significant difference between the two groups. Although there was no control group to determine the effects TXA may have had on VTE development, the latest meta-analysis supports the safety of TXA in joint arthroplasty [18].

We do acknowledge the limitations of our study. First, we did not assess functional factors such as VAS score, knee range of motion, knee swelling, and walking ability. Second, we did not measure serum levels of PAI TXA vs IV TXA, thus limiting our interpretation of its systemic effects. Third, this study excluded patients who would be deemed at the most risk if they were to receive IV TXA. To compare the safety of each modality, we would require a large number of patients and may not be practical to investigate in a prospective, randomized study. Fourth, the timing was also different with IV being given immediately preoperatively and PAI being injected in the later stages of the surgery, which may have made it difficult to identify the local effect of PAI TXA in reducing blood loss. In addition, our study did not involve the determination of the optimal dosage of TXA, which also is a shortage and needs further studies to compare whether different doses of TXA can produce different results.

This randomized controlled trial has demonstrated encouraging results regarding the novel use of TXA as part of a combined peri-articular injection in TKA with a reduction in blood loss versus the more commonly used IV administration of TXA. The use of locally injected TXA is an effective and simple method of reducing blood loss and should be considered as an alternative to IV or topical application in primary TKA.

## Abbreviations

TXA: Tranexamic acid
TKA: Total knee arthroplasty
IV: Intravenous
PAI: Peri-articular injection
IA: Intra-articular injection
TBL: Total blood loss
HBL: Hidden blood loss
